# Effect of neoadjuvant chemotherapy on the immune microenvironment in non–small cell lung carcinomas as determined by multiplex immunofluorescence and image analysis approaches

**DOI:** 10.1186/s40425-018-0368-0

**Published:** 2018-06-06

**Authors:** Edwin R. Parra, Pamela Villalobos, Carmen Behrens, Mei Jiang, Apar Pataer, Stephen G. Swisher, William N. William, Jiexin Zhang, Jack Lee, Tina Cascone, John V. Heymach, Marie-Andrée Forget, Cara Haymaker, Chantale Bernatchez, Neda Kalhor, Annikka Weissferdt, Cesar Moran, Jianjun Zhang, Ara Vaporciyan, Don L. Gibbons, Boris Sepesi, Ignacio I. Wistuba

**Affiliations:** 1 Department of Translational Molecular Pathology, Unit 951, The University of Texas MD Anderson Cancer Center, 2130 West Holcombe Blvd, Houston, TX 77030 USA; 2 Department of Thoracic/Head and Neck Medical Oncology, The University of Texas MD Anderson Cancer Center, Houston, TX USA; 3 Department of Thoracic and Cardiovascular Surgery, Unit 1489, The University of Texas MD Anderson Cancer Center, 1400 Pressler St. Houston, Houston,, TX 77030 USA; 4 Department of Bioinformatics and Computational Biology, The University of Texas MD Anderson Cancer Center, Houston, TX USA; 5 Department of Biostatistics, The University of Texas MD Anderson Cancer Center, Houston, TX USA; 6 Department of Melanoma Medical Oncology, The University of Texas MD Anderson Cancer Center, Houston, TX USA; 7 Department of Anatomical Pathology, The University of Texas MD Anderson Cancer Center, Houston, TX USA

**Keywords:** Tumor compartments, Epithelial compartment, Stromal compartment, Adenocarcinoma, Squamous cell carcinoma, Survival, T cells

## Abstract

**Background:**

The clinical efficacy observed with inhibitors of programed cell death 1/programed cell death ligand 1 (PD-L1/PD-1) in cancer therapy has prompted studies to characterize the immune response in several tumor types, including lung cancer. However, the immunological profile of non–small cell lung carcinoma (NSCLC) treated with neoadjuvant chemotherapy (NCT) is not yet fully characterized, and it may be therapeutically important. The aim of this retrospective study was to characterize and quantify PD-L1/PD-1 expression and tumor-associated immune cells (TAICs) in surgically resected NSCLCs from patients who received NCT or did not receive NCT (non-NCT).

**Methods:**

We analyzed immune markers in formalin-fixed, paraffin-embedded tumor tissues resected from 112 patients with stage II/III NSCLC, including 61 non-NCT (adenocarcinoma [ADC] = 33; squamous cell carcinoma [SCC] = 28) and 51 NCT (ADC = 31; SCC = 20). We used multiplex immunofluorescence to identify and quantify immune markers grouped into two 6-antibody panels: panel 1 included AE1/AE3, PD-L1, CD3, CD4, CD8, and CD68; panel 2 included AE1/AE3, PD1, granzyme B, FOXP3, CD45RO, and CD57.

**Results:**

PD-L1 expression was higher (> overall median) in NCT cases (median, 19.53%) than in non-NCT cases (median, 1.55%; *P* = 0.022). Overall, density of TAICs was higher in NCT-NSCLCs than in non-NCT-NSCLCs. Densities of CD3+ cells in the tumor epithelial compartment were higher in NCT-ADCs and NCT-SCCs than in non-NCT-ADCs and non-NCT-SCCs (*P* = 0.043). Compared with non-NCT-SCCs, NCT-SCCs showed significantly higher densities of CD3 + CD4+ (*P* = 0.019) and PD-1+ (*P* < 0.001) cells in the tumor epithelial compartment. Density of CD68+ tumor-associated macrophages (TAMs) was higher in NCT-NSCLCs than in non-NCT-NSCLCs and was significantly higher in NCT-SCCs than in non-NCT-SCCs. In NCT-NSCLCs, higher levels of epithelial T lymphocytes (CD3 + CD4+) and epithelial and stromal TAMs (CD68+) were associated with better outcome in univariate and multivariate analyses.

**Conclusions:**

NCT-NSCLCs exhibited higher levels of PD-L1 expression and T-cell subset regulation than non-NCT-NSCLCs, suggesting that NCT activates specific immune response mechanisms in lung cancer. These results suggest the need for clinical trials and translational studies of combined chemotherapy and immunotherapy prior to surgical resection of locally advanced NSCLC.

**Electronic supplementary material:**

The online version of this article (10.1186/s40425-018-0368-0) contains supplementary material, which is available to authorized users.

## Background

Tumors grow by using a complex composite system that includes epithelial and stromal cell activation, vessel proliferation, and inflammatory and immune cell activation [1]. In normal situations, T lymphocytes recognize malignant cells as abnormal and activate cytotoxic T lymphocytes through helper T cells at the site, which infiltrate and kill the malignant cells. However, malignant cells have developed sophisticated mechanisms and pathways through which they regulate negative and positive signals, blocking cytotoxic T cell activation and regulatory T cells and thus promoting tumor growth and eventual tumor metastasis [2].

An increasing number of studies as well as clinical trials in the past few years have demonstrated the oncologic effectiveness of antibody inhibitors of immune checkpoints; by inhibiting these checkpoints, these antibodies facilitate release of inhibitory signals and augment the antitumor activity of the immune system. The remarkable clinical efficacy observed with inhibitors of immune checkpoints such as programed cell death 1/programed cell death ligand 1 (PD-L1/PD-1) [3–5] has become increasingly important in studying the role of the immune cell system in controlling tumor growth in various types of cancer.

Various aspects of immune cells, such as type, functional polarization, and local distribution through the tumor, have been shown to influence clinical outcome for cancer patients [6]. Accumulating evidence shows that high densities of mature T lymphocytes, in particular those with cytotoxic function such as CD8+ and natural killer cells, correlate with favorable prognosis, both in terms of recurrence-free survival (RFS) and overall survival (OS), in various cancer types, including lung cancer [7–11]. These findings strongly indicate that a natural immune cell reaction controls the escape of metastatic cells and reduces cancer aggressiveness [12], suggesting that strategies to control and modify the immune cell population are important approaches to cancer therapy. Although neoadjuvant chemotherapy (NCT) for cancer has historically been considered immunosuppressive, it is now accepted that certain chemotherapy agents, such as paclitaxel, cisplatin, gemcitabine, and carboplatin, can regulate and modulate antitumor immunity [13–17]. Chemotherapy has a potential to trigger immune activation by inducing immunogenic cell death and subsequent tumor-associated neoantigen release, which in turn activates antigen-presenting cells (APCs) such as tumor-associated macrophages (TAMs) and dendritic cells through Toll-like receptors [18–21]. For this study, therefore, we hypothesized that NCT influences anticancer response by favorably altering the immune microenvironment.

The aim of this retrospective study was to identify and quantify chemotherapy-induced changes in the immune microenvironment, including PD-L1/PD-1 expression, in the tumor and tumor-associated immune cells (TAICs) using a multiplex immunofluorescence methodology [22]. We used this approach to compare surgically resected non–small cell lung carcinoma (NSCLC) specimens from patients who received NCT with specimens from patients who underwent primary surgical resection (non-NCT).

## Methods

### Cases and specimens

Formalin-fixed, paraffin-embedded (FFPE) histologic sections of NSCLCs were prospectively identified from primary tumors resected from 112 patients who underwent surgery with curative intent between January 1, 1997, and December 31, 2012, at The University of Texas MD Anderson Cancer Center. Of the 112 patients, 61 underwent primary surgical resection (non-NCT group); 33 of the patients in this group had adenocarcinoma (non-NCT-ADC) and 28 had squamous cell carcinoma (non-NCT-SCC). The comparison group consisted of 51 patients who received NCT prior to surgical resection. This group comprised 31 with adenocarcinoma (NCT-ADC) and 20 with squamous cell carcinoma (NCT-SCC). Tumor stage was classified according to the systems of the World Health Organization, 4th edition [23], and the International Association for the Study of Lung Cancer, 7th edition [24]. Clinicopathologic information, including demographic data, smoking status (current, former, or never), tumor size before NCT (according to image scanning tomography reports) and after NCT (pathologic report), type of NCT used, adjuvant treatment, and follow-up information (RFS and OS), was retrieved from patients’ electronic medical records. The study received approval from the MD Anderson Cancer Center Institutional Review Board; written informed consent was required of and obtained from all patients.

### Multiplex immunofluorescence staining

Manual multiplex immunofluorescence (mIF) staining was performed in 4-μm sequential histologic tumor sections obtained from representative FFPE tumor blocks by using the Opal 7-Color fIHC Kit (PerkinElmer, Waltham, MA). The stained slides were scanned by a Vectra multispectral microscope (PerkinElmer) [22]. The immunofluorescence (IF) markers used were grouped into two 6-antibody panels: panel 1 consisted of pancytokeratin AE1/AE3 (epithelial cell marker; dilution 1:300; Dako, Carpinteria, CA), PD-L1 (clone E1L3N, dilution 1:100; Cell Signaling Technology, Beverly, MA), CD3 (T lymphocyte marker; dilution 1:100; Dako), CD4 (helper T cell marker; Novocastra clone 4B12, dilution 1:80; Leica Biosystems, Buffalo Grove, IL), CD8 (cytotoxic T cell marker; clone C8/144B, dilution 1:20; Thermo Fisher Scientific, Waltham, MA), and CD68 (macrophage marker; clone PG-M1, dilution 1:450; Dako). Panel 2 consisted of pancytokeratin AE1/AE3 (dilution 1:300; Dako), PD-1 (clone EPR4877–2, dilution 1:250; Abcam, Cambridge, MA), granzyme B (cytotoxic lymphocyte marker; clone F1, ready to use; Leica Biosystems), FOXP3 (regulatory T cell marker; clone 206D, dilution 1:50; BioLegend, San Diego, CA), CD45RO (memory T cell marker; clone UCHL1, ready to use; Leica Biosystems), and CD57 (natural killer cell marker; clone HNK-1, dilution 1:40; BD Biosciences, San Jose, CA).

Primary antibody was visualized by using tyramide signal amplification linked to a specific fluorochrome from the Opal 7-Color fIHC Kit for each primary antibody. A stripping procedure, based on the EZ Retriever microwave (BioGenex, Fremont, CA), was performed for each consecutive antibody staining. In parallel, uniplex IF was used with each individual antibody and with the same fluorochrome used in the mIF to create the spectral library in human tonsil FFPE tissues used in the multispectral analysis [25]. Human tonsil FFPE tissues were also used with and without primary antibodies as positive and negative (autofluorescence) controls, respectively. The mIF- and uniplex IF-stained slides were scanned with a Vectra 3.0 microscope system (PerkinElmer) under fluorescent illumination. From each slide, Vectra automatically captured the fluorescent spectra from 420 nm to 720 nm at 20-nm intervals with the same exposure time and then combined the captured images to create a single stack image that retained the particular spectral signature of all IF markers [25]. After the specimens were scanned at low magnification (×10), five individual fields (669×500 μm each) in the tumor area were examined with a Phenochart 1.0.4 (PerkinElmer) viewer so that they could be scanned at high resolution (×20) to capture various elements of tumor heterogeneity. Histologic assessment of each tumor area ensured that clusters of malignant cells were included in the selected area and that each area from panel 1 overlapped with the sequential tissue from panel 2.

### Multispectral analysis

Tumor multispectral images containing PD-L1 and TAICs, including tumor-infiltrating lymphocyte (TIL) markers, were analyzed in two compartments: the epithelial compartment, defined as malignant cell nests, and the stromal compartment, characterized by the fibrous tissue present between malignant cells, as previously described [9]; these compartments were identified by applying the tissue segmentation tool of the InForm 2.1.0 software (PerkinElmer) (Additional file 1: Figure S1). The individual cells (defined by nuclei [DAPI] staining) identified by the cell segmentation tool were subjected to the phenotyping pattern recognition learning algorithm tool to characterize co-localization of the various cell populations [26] using panel 1 and panel 2 labeling. Panel 1 (Additional file 2: Figure S2) labeling was as follows: malignant cells expressing PD-L1 (AE1/AE3 + PD-L1+); T lymphocytes (CD3; pan T-cell marker including helper T cells CD3 + CD4+, cytotoxic T cells CD3 + CD8+, and other CD3+ T cells); helper T cells (CD3 + CD4+); cytotoxic T cells (CD3 + CD8+); TAMs (CD68+); and TAMs expressing PD-L1 (CD68 + PD-L1+). Panel 2 (Additional file 3: Figure S3) labeling was as follows: memory cells (CD45RO; including memory/natural killer cells CD45RO + CD57 + granzyme B−, memory/regulatory cells CD45RO + FOXP3+, memory antigen experienced cells CD45RO + PD-1+, and other CD45RO+ cells); memory/regulatory cells (CD45RO + FOXP3+); memory antigen experienced cells (CD45RO + PD-1+); activated natural killer cells (CD57 + granzyme B + CD45RO−); and antigen experienced cells (PD-1; including PD-1 + CD45RO+ and other PD-1+ cells). The analysis with each panel created a comprehensive cell-by-cell identification report of expression of the antibody markers in both compartments. The individual cell report created by InForm was processed by Spotfire software (TIBCO, PerkinElmer) to create a final data report expressing the results as number of cells/mm^2^ from each individual cell phenotyping population as well as the percentage of TAMs and malignant cells expressing PD-L1 for the statistical analysis.

### Statistical methods

Statistical analyses were carried out with the R software program (version 3.3.0, released May 2016; Vienna, Austria; URL https://www.R-project.org/). Expression greater than the median percentage of membranous PD-L1 expression in malignant cells was considered positive; based on this and on measured cell densities per mm^2^, we divided patients into two groups, high and low, relative to the median number of TAICs per mm^2^. Differences between groups for all parameters were determined by using the Mann–Whitney *U* test (unpaired, nonparametric, two-tailed), except for RFS and OS studies, in which the log rank test was used. RFS was defined as the interval from surgery to recurrence or last contact, and OS was defined as the interval from surgery to death or last contact. As described previously by Pataer and colleagues [27], the hematoxylin and eosin–stained slides from NCT patients were examined to determine the percent tumor viability and its influence on survival at a 10% cutoff. Multivariate Cox proportional hazard regression models and logistic regression models were utilized to study the variables significant in the univariate analysis and their association with outcome.

## Results

### Clinicopathologic characteristics

**Fig. 1:**
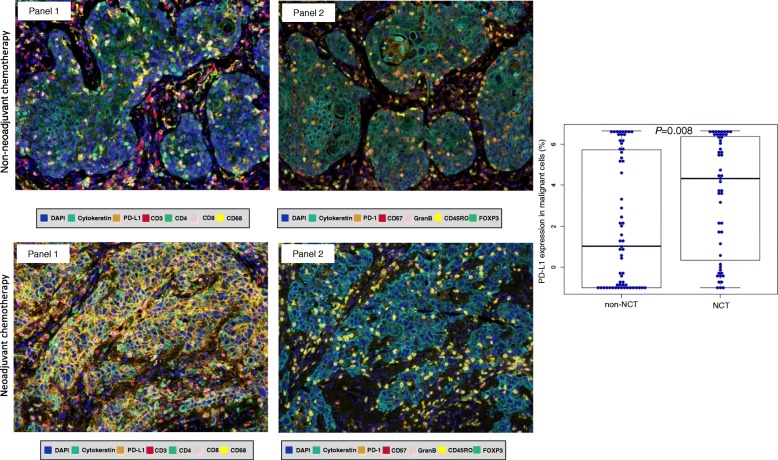
Representative multiplex immunofluorescences and PD-L1 expression in non-NCT and NCT. (Left) Multiplex immunofluorescence images of representative NSCLC tumor sections analyzed for panel 1 and panel 2 markers: upper images are from the group that did not receive neoadjuvant chemotherapy (non-NCT), while the lower images are from the group that did receive NCT. The images reflect the variations in cell phenotypes observed in these cases. (Right) Box plot showing that PD-L1 expression by malignant NSCLC cells was higher in the group that received NCT than in the non-NCT group. Images ×200

Using mIF and image analysis approaches, we evaluated the immune microenvironment of NSCLCs from patients who did or did not receive NCT (Fig. 1). Clinicopathologic features and chemotherapy treatment data are summarized in Table 1. The median interval between completion of NCT and surgical resection was 35 days (min/max, 17/75 days). The median numbers of malignant cells expressing PD-L1+ and the TAIC densities in the non-NCT and NCT groups are shown in Table 2. We identified no significant correlations between clinicopathologic features and malignant cell expression of PD-L1+ or TAIC density in either the non-NCT or the NCT group, nor did we observe differences related to chemotherapy regimen or interval between surgical resection and completion of NCT.

**Table 1:** Characteristics of NSCLC patients who received neoadjuvant chemotherapy (NCT) or did not receive NCT (non-NCT) (*N* = 112)

		N (%)	
Characteristic	Category	non-NCT (*n* = 61)	NCT (*n* = 51)
Age	Median	62 years	65 years
Sex	Female	22 (36)	22 (43)
	Male	39 (64)	29 (57)
Tobacco history	No	3 (5)	6 (12)
	Yes	58 (95)	45 (88)
Smoking status	Never	3 (5)	6 (12)
	Former	28 (46)	14 (28)
	Current	30 (49)	31 (60)
Tumor size pretreatment (CT scan)	Median	–	4.50 cm
Tumor size posttreatment (Pathology)^a^	Median	4.50 cm	4.10 cm
Tumor status^b^	T_1_	4 (6)	5 (10)
	T_2_	25 (41)	23 (44)
	T_3_	23 (38)	13 (26)
	T_4_	9 (15)	10 (20)
Nodal status^b^	N_0_	10 (16)	7 (16)
	N_1_	18 (29)	11 (18)
	N_2_	33 (54)	33 (66)
AJCC stage^b^	II	7 (12)	5 (8)
	III	54 (88)	46 (92)
Histology	ADC	33 (54)	31 (60)
	SCC	28 (46)	20 (40)
Neoadjuvant chemotherapy	Carboplatin/paclitaxel	–	23 (45)
	Carboplatin/pemetrexed	–	10 (20)
	Cisplatin/others^c^	–	18 (35)
Adjuvant therapy^d^	No	22 (36)	11 (20)
	Yes	39 (64)	32 (64)
Vital status	Alive	24 (39)	13 (26)
	Dead	37 (61)	38 (74)
Overall survival	Median	34 months	21 months

ADC, adenocarcinoma; SCC, squamous cell carcinoma

^a^by pathology report

^b^by International Association for the Study of Lung Cancer classification system

^c^Other chemotherapies such as gemcitabine or docetaxel

^d^Adjuvant therapy unknown in 8 cases from NCT group

**Table 2:** Median densities of various immune marker–expressing cells in NSCLCs from patients who received neoadjuvant chemotherapy (NCT) or did not receive NCT (non-NCT) (*N* = 112)

Markers	non-NCT (*n* = 61)	NCT (*n* = 51)	*P* ^a^
	Median Cell Density (cells/mm^2^)		
Panel 1			
MCs (AE1/AE3+)	3559.79	3269.35	0.615
MCs PD-L1+	34.37	574.58	*0.022*
CD3+	903.21	1501.99	*0.021*
CD3 + CD4+	671.00	1031.88	*0.040*
CD3 + CD8+	156.38	276.08	0.588
CD68+	298.80	609.36	0.059
CD68 + PD-L1+	194.46	307.32	0.122
Panel 2			
MCs (AE1/AE3+)	3536.02	2970.70	0.157
CD45RO+	668.75	1180.26	0.290
CD45RO + CD57 + granzyme B−	679.09	965.58	0.147
CD45RO + PD-1+	153.73	443.04	*< 0.001*
CD45RO + FOXP3+	5.38	8.37	0.427
CD57 + granzymeB+	6.87	20.32	*< 0.001*
PD-1+	336.02	795.21	*< 0.001*

*MC* malignant cells

^a^Mann Whitney U test

### PD-L1 expression by malignant cells higher in NCT-treated tumors

Density of malignant cells expressing PD-L1 (AE1/AE3 + PD-L1+) was higher in NCT-treated tumors (median, 574.58 cells/mm^2^) than in non-NCT tumors (median, 34.37 cells/mm^2^, *P* = 0.022) (Table 2). The percentage of malignant cells expressing PD-L1 also was higher in NCT-treated tumors (median, 19.53%) than in non-NCT tumors (median, 1.55%; *P* = 0.008) (Fig. 1). Although both ADCs and SCCs in the NCT group showed higher PD-L1 expression by malignant cells, only NCT-ADC showed significantly higher density and percentage of malignant cells expressing PD-L1 (median, 362.92 cells/mm^2^, 11.10%) than their non-NCT counterparts (median, 13.45 cells/mm^2^, 0.29%; *P* = 0.007 and *P* = 0.008, respectively) (Table 3).

**Table 3:** Median densities of various immune marker–expressing cells in NSCLCs from patients who received neoadjuvant chemotherapy (NCT) or did not receive NCT (non-NCT), by tumor histology (*N* = 112)

Markers	ADC (*n* = 64)		*P* ^a^	SCC (*n* = 51)		*P* ^a^
	non-NCT	NCT		non-NCT	NCT	
	Median Cell Density (cells/mm^2^)			Median Cell Density (cells/mm^2^)		
Panel 1						
MCs (AE1/AE3+)	3135.42	3043.84	0.841	4125.37	3641.55	0.554
MCs PD-L1+	13.45	362.92	*0.007*	317.91	944.99	0.593
CD3+	916.14	1406.72	0.133	811.47	1825.85	0.070
CD3 + CD4+	671.49	997.47	0.170	650.63	1360.68	0.072
CD3 + CD8+	157.39	212.85	0.647	155.34	358.93	0.842
CD68+	298.80	591.42	0.430	321.12	705.60	*0.040*
CD68 + PD-L1+	196.71	222.96	0.600	172.72	357.54	0.090
Panel 2						
MCs (AE1/AE3+)	3134.08	2596.71	0.299	4528.99	3703.28	0.352
CD45RO+	623.31	595.51	0.484	723.46	1356.35	*< 0.001*
CD45RO + CD57 + granzyme B−	240.95	332.13	*0.008*	118.68	395.21	*0.013*
CD45RO + PD1+	170.47	243.04	*0.016*	112.40	380.26	*0.007*
CD45RO + FOXP3+	7.17	7.17	0.448	4.18	9.56	0.073
CD57 + granzyme B+ CD45RO−	4.26	40.65	*< 0.001*	9.86	9.86	0.925
PD-1+	426.90	718.68	*0.014*	273.84	1314.49	*< 0.001*

*ADC* adenocarcinoma, *SCC* squamous cell carcinoma, *MC* malignant cells

^a^Mann Whitney U test

### TAIC densities higher in NCT-treated tumors

**Fig. 2:**
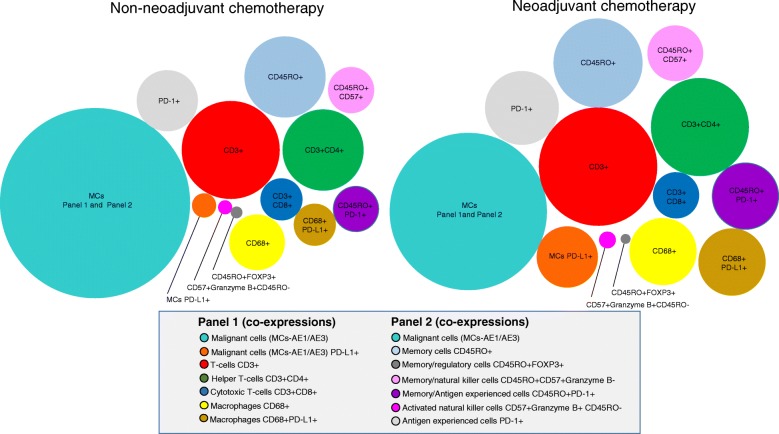
Representative figure compared phenotypes between non-NCT and NCT. Graphic representation of relative densities of different cell phenotypes detected by analysis with panel 1 and 2 markers in NSCLCs that were treated or not treated with neoadjuvant chemotherapy (NCT). Overall, the numbers of various immune cell phenotypes were higher in the group that received NCT than in the non-NCT group

As shown in Table 2**,** Fig. 2 and Additional file 4: Figure S4, the densities of TAICs of various phenotypes were higher overall in NCT tumors than in non-NCT tumors. The number of T lymphocytes (CD3+) was significantly higher in NCT tumors than in non-NCT tumors (*P* = 0.021). Furthermore, the densities of T lymphocytes (CD3+), helper T cells (CD3 + CD4+), activated natural killer cells (CD57 + granzyme B + CD45RO−), memory antigen experienced cells (CD45RO + PD-1+), and antigen experienced (PD-1+) cells were higher in NCT tumors than in non-NCT tumors (between *P =* 0.040 and *P* < 0.001). Density of TAMs (CD68+) was also higher in NCT tumors than in non-NCT tumors (*P* = 0.059). Although the densities of TAICs overall were higher in NCT-ADCs and NCT-SCCs than in non-NCT-ADCs and non-NCT-SCCs, as shown in Table 3, the NCT-ADC tumors showed significantly higher densities of activated natural killer cells (CD57 + granzyme B + CD45RO−), memory/natural killer T-cells (CD45RO + CD57 + granzyme B−), memory antigen experienced cells (CD45RO + PD-1+), and antigen experienced (PD-1+) cells than non-NCT-ADCs (*P* < 0.001, *P* = 0.008, *P* = 0.016, *P* = 0.014, respectively), while the NCT-SCCs showed significantly higher densities of memory cells (CD45RO+), memory/natural killer T cells (CD45RO + CD57 + granzyme B−), memory antigen experienced cells (CD45RO + PD-1+), antigen experienced (PD-1+) cells, and TAMs (CD68+) than non-NCT-SCCs (between *P* = 0.040 and *P* < 0.001).

### TAIC densities higher in both epithelial and stromal compartments of NCT tumors

The TAIC density differences between non-NCT and NCT tumors were independent of histology and of compartment. As shown in Additional file 5: Table S1 and Additional file 6: Figure S5, the densities of TAICs were higher overall in the stromal compartments of non-NCT and NCT tumors than in their respective epithelial compartments. In the epithelial compartments, the densities of T lymphocytes (CD3+), helper T cells (CD3 + CD4+), activated natural killer cells (CD57 + granzyme B + CD45RO−), memory/natural killer T cells (CD45RO + CD57 + granzyme B−), memory antigen experienced cells (CD45RO + PD-1+), antigen experienced (PD-1+) cells, and TAMs (CD68+) were significantly higher in the NCT group than in the non-NCT group. Density of TAMs (CD68+) expressing PD-L1+ was significantly higher in the epithelial compartments of NCT tumors than in those of non-NCT tumors (between *P* = 0.049 and *P* < 0.001).

Density of memory/regulatory cells (CD45RO + FOXP3+) in the epithelial compartment was significantly lower in NCT tumors than in non-NCT tumors (*P* = 0.092). However, densities of T lymphocytes (CD3+) and activated natural killer cells (CD57 + granzyme B + CD45RO−) in the stromal compartment were significantly higher in the NCT tumors than in non-NCT tumors (*P* = 0.029 and *P* = 0.002, respectively). As in the epithelial compartment, density of memory/regulatory cells (CD45RO + FOXP3+) in the stromal compartment was lower in NCT tumors than in non-NCT tumors, but the difference was not significant (*P* = 0.060).

When the analysis included both tumor histology and compartment, as shown in Additional file 7: Table S2, cell densities were higher overall in both compartments of NCT-ADCs and NCT-SCCs than in those of non-NCT-ADCs and non-NCT-SCCs. Important and significant differences were observed in various cell phenotypes: in NCT-ADCs, the densities of activated natural killer cells (CD57 + granzyme B + CD45RO−) in both the epithelial and stromal compartments were significantly higher than those in non-NCT-ADCs (*P* = 0.001 and *P* = 0.001, respectively). However, densities of memory/regulatory cells (CD45RO + FOXP3+) in both epithelial and stromal compartments were lower in NCT-ADCs than in non-NCT-ADCs (*P* = 0.085 and *P* = 0.001, respectively), but the difference was significant only in the stromal compartment. In the epithelial compartments of SCCs, the densities of T lymphocytes (CD3+), helper T cells (CD3 + CD4+), antigen experienced (PD-1+) cells, and TAMs (CD68+) were higher in the NCT group than in the non-NCT group (*P* = 0.023, *P* = 0.019, *P* < 0.001, and *P* = 0.016, respectively). In the stromal compartments of SCCs, the density of antigen experienced (PD-1+) cells was higher in NCT tumors than in non-NCT tumors (*P* = 0.015).

### Inflammatory cell–modulated prognostic correlations in NCT patients

**Fig. 3:**
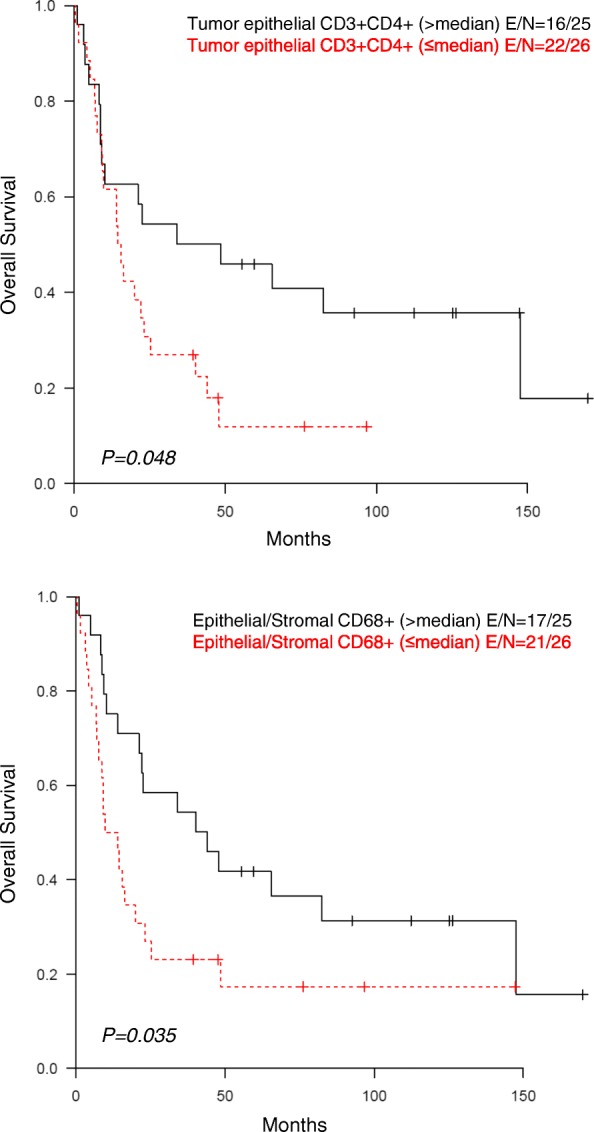
Univariate analysis in NCT. Kaplan-Meier analysis of patients with NSCLC who received NCT showed longer survival among those with a higher density of helper T cells (CD3 + CD4+) in the tumor epithelial compartment and among those with a higher density of CD68+ tumor-associated macrophages in the tumor epithelial and stromal compartments

To identify the contribution of each immune cell subpopulation to the biological behavior of NCT-treated lung tumors, we analyzed their impact on long-term survival. The main observed differences were for epithelial helper T cells (CD3 + CD4+) and epithelial/stromal TAMs (CD68+), which have been previously linked to better prognosis. At a tumor viability cutoff of 10% in NCT, no prognostic difference was observed. In the entire cohort of NCT-treated patients (ADC and SCC), OS was longer, based on univariate analysis, in patients with higher densities of helper T cells (CD3 + CD4+; *P* = 0.048) and TAMs (CD68+; *P* = 0.035) (Fig. 3). Logistic regression multivariate models incorporating tumor stage corroborated the association between survival and higher epithelial and stromal densities of TAMs (CD68+) (*P* = 0.044; hazard ratio [HR], 0.506; 95% confidence interval [CI], 0.261–0.982) and higher epithelial density of helper T cells (CD3 + CD4+) (*P* = 0.097; HR, 0.547; 95% CI, 0.269–1.114) (Additional file 8: Table S3**)**.

## Discussion

In this study, mIF and image analysis were used to evaluate both PD-L1 expression and densities of TAIC populations via high-throughput analysis of tumor epithelial and stromal compartments in NSCLCs treated or not treated with NCT. Utilizing the median PD-L1 expression value in malignant cells as the cut-off for positive expression, we observed higher densities and percentages of PD-L1+ malignant cells in NCT-treated tumors than in non-NCT tumors. Similarly, utilizing the median TAIC density as the cut-off value, we observed higher densities of TAICs in NCT-treated tumors than in non-NCT tumors. Among NCT-treated patients, Kaplan-Meier analysis showed better prognosis for patients with higher-than-median density of helper T cells (CD3 + CD4+) in the tumor epithelial compartment and higher-than-median densities of TAMs (CD68+) in the tumor epithelial and stromal compartments than for patients with lower-than-median densities of these cells.

To our knowledge, this is the first study that used mIF to analyze and compare large panels of immune-profiling markers in NSCLC tissues that had or had not been treated with NCT. Similar to some studies in urothelial [28], thymic [29], ovarian [30], lung [31], and head and neck [32] cancers treated with NCT, we found higher densities and percentages of malignant cells expressing PD-L1 in NCT-treated tumors, independent of the NCT regimen, than in non-NCT tumors, suggesting that PD-L1 expression can be increased by NCT, potentially through activation of immune-related pathways such as IFNα, IFNγ, STAT3, and TNFα [33]. Previous research has demonstrated that immunogenic death of malignant cells induced by various chemotherapy regimens [34] can enhance the activation of APCs, which in turn activates TILs and their production of IFN-γ, which subsequently increases the expression of PD-L1 by malignant cells [33]. Although exposure of tumor cell lines to various chemotherapy agents [35, 36] and some mechanisms of action of chemotherapy agents [37–39] can increase PD-L1 expression in cancer cells, the exact mechanisms by which chemotherapy drugs induce this increase remain unknown.

TAICs play an important role in anticancer immune surveillance [40]. TAICs, including TILs and TAMs, are involved in the anticancer immune response, and it is known that TILs, which are generally represented by cytotoxic T cells (CD3 + CD8+) and helper T cells (CD3 + CD4+), along with natural killer cells [41, 42], have an important role in anticancer immunity. Malignant cells are usually killed by several pathways that induce cell apoptosis, orchestrated by a successful cytotoxic attack mediated by APCs such as TAMs or dendritic cells [43]. The significantly higher numbers of T lymphocytes, helper T cells, activated natural killer cells, memory/natural killer cells, and memory antigen experienced cells observed in NCT-treated specimens suggest that, in general, cytotoxic chemotherapy may induce tumor immunogenicity through the release of neoantigens from apoptotic malignant cells [44].

It has been reported that tumors that otherwise express low levels of antigens, when treated with chemotherapy, undergo sufficient release of antigens to sensitize stromal cells for destruction by cytotoxic T cells [43]. In the NCT-treated group, amounts of specific TILs such as helper T cells (CD3 + CD4+) and activated natural killer cells (CD57 + granzyme B + CD45RO−) were increased in both the epithelial and stromal compartments. This suggests that chemotherapy induces recruitment of inflammatory cells against malignant cells, with especially significantly higher expression of helper T cells (CD3 + CD4+) in the epithelial compartment of SCCs and activated natural killer cells (CD57 + granzyme B + CD45RO−) in the epithelial compartment of ADCs. Helper T cells (CD3 + CD4+) are important for initiating and maintaining anticancer immune responses; in their absence, specific cytotoxic T lymphocytes can become lethargic [45] or depleted [46]. Helper T cells are also essential for inducing transformation of cytotoxic T cells into long-lived functional effector cells [47].

Activated natural killer cells (CD57 + granzyme B+ CD45RO−) have a high cytotoxic potential [48], suggesting that the activation of these cells could have important therapeutic implications. Overall, quantities of memory/regulatory T-cells (CD45RO + FOXP3+) and antigen experienced cells (PD-1+) also were increased in NCT-treated cases, predominantly SCCs. When we analyzed data by tumor compartment, we found that quantity of memory/regulatory T cells (CD45RO + FOXP3+) was significantly lower in the stromal compartment of NCT-ADCs than in the stromal compartment of non-NCT-ADCs, suggesting that chemotherapy can modulate phenotype by tumor tissue compartment. The reasons for this discrepancy are still unclear, but the variety of chemotherapy regimens cannot be ruled out, since immune effectiveness differs among different drugs and histologies.

Overall, NCT-treated tumors showed a higher density of TAMs than non-NCT tumors. TAMs (CD68+) were significantly more abundant in NCT-SCCs, particularly in the epithelial compartment in direct contact with malignant cells. Like Hiraoka and colleagues [11], we found that higher density of epithelial helper T cells (CD3 + CD4+) correlated with a better prognosis in univariate analysis; however, this association was not confirmed in the multivariate analysis. Both our univariate and multivariate analyses did show, though, that higher densities of epithelial and stromal TAMs (CD68+) correlated with better survival in NCT-treated NSCLC patients. As suggested by Blankenstein [49], tumor suppression factors are meditated by activation of TAM class M1 in the tumor epithelial and stromal compartments and helper T cells (CD3 + CD4+) in the tumor epithelial compartment. To further define the importance of TAMs in NCT-treated tumors, future studies should focus on the subtypes of TAMs, since recent studies [50] suggest that TAMs polarize to M1 (anti-tumorigenic) or M2 (contributing to carcinogenesis) subtypes and thus can exert differential effects in the tumor microenvironment.

Our study has several limitations. The number of NCT-treated patients is rather small, and the cohorts consist mainly of patients with stage III disease. Although the variability of the chemotherapy regimens is a potential confounder of our results, no differences were observed in the various regimens’ impacts on the immune microenvironment. The cohort of stage III patients who did not undergo induction chemotherapy (non-NCT) likely comprised patients with incidentally found stage III (N2) lung cancer in pathologic specimens, because our preference since publication of Roth et al. [51] has been to administer chemotherapy prior to surgical resection for stage III lung cancers. This group of patients was likely clinically different from the group that received NCT. Further studies are needed to validate our findings, to establish clinically meaningful reference values for TAICs in the tumor and peritumoral compartments based on mIF techniques, and to sort out the details of the mechanisms by which chemotherapy induces a potentially favorable microenvironment for the administration of checkpoint inhibitor immunotherapy.

## Conclusions

Patients with NSCLC treated with NCT exhibited higher levels of PD-L1+ malignant cells and TAIC than patients who underwent upfront tumor resection without NCT. In patients who underwent NCT, those with higher abundance of helper T cells and TAMs survived longer, suggesting that these cells may important in chemotherapy response. Together, our findings suggest that chemotherapy may activate immune response mechanisms such as IFNα, IFNγ, STAT3, and TNFα in lung cancer, which may generate a favorable tumor microenvironment for subsequent response to checkpoint immunotherapy.

## Additional files

Additional file 1: **Figure S1.** Representative multiplex immunofluorescence (mIF) workflow, showing different steps in the process of analysis. (A) Vectra 3.0 multispectral microscope used for the analysis. (B) Phenochart 1.0.4 software showing an image scanning at ×10 and selection of five intratumoral areas (669×500 μm each) for further analysis. (C) mIF image scanning at ×20 and viewed in the InForm 2.1.3 image analysis software. (D) Composed image showing the different phenotypes with panel 1. (E) Tumor segmentation (epithelial and stromal compartments) using the InForm software. (E) Cell segmentation to characterize individual cells using DAPI as a counterstaining marker, and cell phenotyping identifying different subgroups of cells supervised by a pathologist. (PPTX 6362 kb)

Additional file 2: **Figure S2.** Characterization of immune cell phenotyping using marker panel 1. Cell phenotypes were defined as malignant cells expressing PD-L1 (AE1/AE3 + PD-L1+), T lymphocytes (CD3; pan T-cell marker including helper T cells CD3 + CD4+, cytotoxic T cells CD3 + CD8+, and other CD3+ T cells), helper T cells (CD3 + CD4+), cytotoxic T cells (CD3 + CD8+), tumor-associated macrophages (TAMs; CD68+), and TAMs expressing PD-L1 (CD68 + PD-L1+). Each type of cell is shown as individual images by marker and by a composite image. (PPTX 102487 kb)

Additional file 3: **Figure S3.** Characterization of immune cell phenotyping using marker panel 2. Cell phenotypes were defined as memory cells (CD45RO, including memory/natural killer cells CD45RO + CD57 + granzyme B−, memory/regulatory cells CD45RO + FOXP3+, memory antigen experienced cells CD45RO + PD-1+, and other CD45RO+ cells), memory/regulatory cells (CD45RO + FOXP3+), memory antigen experienced cells (CD45RO + PD-1+), activated natural killer cells (CD57 + granzyme B + CD45RO−), and antigen experienced cells (PD-1, including PD-1 + CD45RO+ and other PD-1+ cells). Each type of cell is shown as individual images by marker and by a composite image. (PPTX 103717 kb)

Additional file 4: **Figure S4.** Multiplex immunofluorescence images showing densities of various tumor-associated immune cell phenotypes as determined by panel 1 and panel 2 markers from representative NSCLCs treated with neoaduvant chemotherapy (NCT) or not treated with NCT (non-NCT). Numbers of T lymphocytes (CD3+), helper T cells (CD3 + CD4+), tumor-associated macrophages (TAM; CD68+), activated natural killer cells (CD57 + granzyme B + CD45RO−), memory antigen experienced cells (CD45RO + PD-1+), and antigen experienced PD-1+ cells as well as PD-L1+ malignant cells were higher in the NCT group than in the non-NCT group. Images ×200. (PPTX 29362 kb)

Additional file 5: **Table S1.** Median densities of tumor-associated immune cells in NSCLCs of patients who received neoadjuvant chemotherapy (NCT) or did not receive NCT (non-NCT), by tumor compartment (*N* = 112) (DOCX 21 kb)

Additional file 6: **Figure S5.** Multiplex immunofluorescence images showing densities of various tumor-associated immune cell phenotypes (TAICs) as determined by panel 1 and panel 2 markers from the stromal and epithelial compartments of representative NSCLCs treated with neoadjuvant chemotherapy (NCT) or not treated with NCT (non-NCT). In general, densities of TAICs were higher in the stromal compartments than in their respective epithelial compartments in both NCT and non-NCT tumors. Overall, density of tumor-associated macrophages (TAMs; CD68+) was higher in NCT tumors than in non-NCT tumors, and density of memory/regulatory cells (CD45RO + FOXP3+) was lower in the NCT group than in the non-NCT group. Images ×200. (PPTX 15137 kb)

Additional file 7: **Table S2.** Median densities of tumor-associated immune cells in NSCLCs from patients who received neoadjuvant chemotherapy (NCT) or did not receive NCT (non-NCT), by tumor compartment and histology (*N* = 112). (DOCX 23 kb)

Additional file 8: **Table S3.** Multivariate survival analysis (Cox regression model) of effects on survival of (A) tumor-associated macrophages (TAMs; CD68+) and (B) helper T cells (CD3 + CD4+) controlled by tumor stage in NSCLCs from patients who received neoadjuvant chemotherapy (*N* = 51). (DOCX 18 kb)

## Abbreviations

ADC: Adenocarcinoma
APCs: Antigen-presenting cells
FFPE: Formalin-fixed, paraffin-embedded
IF: Immunofluorescence
mIF: Multiplex immunofluorescence
NCT: Neoadjuvant chemotherapy
non-NCT: Non-neoadjuvant chemotherapy
NSCLC: Non–small cell lung carcinoma
OS: Overall survival
PD-L1/PD-1: Programmed cell death 1/programmed cell death ligand 1
RFS: Recurrence-free survival
SCC: Squamous cell carcinoma
TAICs: Tumor-associated immune cells
TAMs: Tumor-associated macrophages
TILs: Tumor-infiltrating lymphocytes

## References

[1] Coussens LM, Werb Z (2002). Inflammation and cancer. Nature.

[2] Igney FH, Krammer PH (2002). Immune escape of tumors: apoptosis resistance and tumor counterattack. J Leukoc Biol.

[3] Hersey P, Gowrishankar K (2015). Pembrolizumab joins the anti-PD-1 armamentarium in the treatment of melanoma. Future Oncol.

[4] Ribas A, Tumeh PC (2014). The future of Cancer therapy: selecting patients likely to respond to PD1/L1 blockade. Clin Cancer Res.

[5] Gettinger SN, Horn L, Gandhi L, Spigel DR, Antonia SJ, Rizvi NA (2015). Overall survival and long-term safety of Nivolumab (anti-programmed death 1 antibody, BMS-936558, ONO-4538) in patients with previously treated advanced non-small-cell lung Cancer. J Clin Oncol.

[6] Fridman WH, Pages F, Sautes-Fridman C, Galon J (2012). The immune contexture in human tumours: impact on clinical outcome. Nat Rev Cancer.

[7] Kawai O, Ishii G, Kubota K, Murata Y, Naito Y, Mizuno T (2008). Predominant infiltration of macrophages and CD8(+) T cells in cancer nests is a significant predictor of survival in stage IV nonsmall cell lung cancer. Cancer.

[8] Ruffini E, Asioli S, Filosso PL, Lyberis P, Bruna MC, Macri L (2009). Clinical significance of tumor-infiltrating lymphocytes in lung neoplasms. Ann Thorac Surg.

[9] Wakabayashi O, Yamazaki K, Oizumi S, Hommura F, Kinoshita I, Ogura S (2003). CD4+ T cells in cancer stroma, not CD8+ T cells in cancer cell nests, are associated with favorable prognosis in human non-small cell lung cancers. Cancer Sci.

[10] Al-Shibli KI, Donnem T, Al-Saad S, Persson M, Bremnes RM, Busund LT (2008). Prognostic effect of epithelial and stromal lymphocyte infiltration in non-small cell lung cancer. Clin Cancer Res.

[11] Hiraoka K, Miyamoto M, Cho Y, Suzuoki M, Oshikiri T, Nakakubo Y (2006). Concurrent infiltration by CD8+ T cells and CD4+ T cells is a favourable prognostic factor in non-small-cell lung carcinoma. Br J Cancer.

[12] Dunn GP, Bruce AT, Ikeda H, Old LJ, Schreiber RD (2002). Cancer immunoediting: from immunosurveillance to tumor escape. Nat Immunol.

[13] Bracci L, Schiavoni G, Sistigu A, Belardelli F (2014). Immune-based mechanisms of cytotoxic chemotherapy: implications for the design of novel and rationale-based combined treatments against cancer. Cell Death Differ.

[14] Wu X, Feng QM, Wang Y, Shi J, Ge HL, Di W (2010). The immunologic aspects in advanced ovarian cancer patients treated with paclitaxel and carboplatin chemotherapy. Cancer Immunol Immunother.

[15] Chen CA, Ho CM, Chang MC, Sun WZ, Chen YL, Chiang YC (2010). Metronomic chemotherapy enhances antitumor effects of cancer vaccine by depleting regulatory T lymphocytes and inhibiting tumor angiogenesis. Mol Ther.

[16] Rettig L, Seidenberg S, Parvanova I, Samaras P, Curioni A, Knuth A (2011). Gemcitabine depletes regulatory T-cells in human and mice and enhances triggering of vaccine-specific cytotoxic T-cells. Int J Cancer.

[17] Weir GM, Liwski RS, Mansour M (2011). Immune modulation by chemotherapy or immunotherapy to enhance cancer vaccines. Cancers (Basel).

[18] Ghiringhelli F, Apetoh L, Tesniere A, Aymeric L, Ma Y, Ortiz C (2009). Activation of the NLRP3 inflammasome in dendritic cells induces IL-1beta-dependent adaptive immunity against tumors. Nat Med.

[19] Apetoh L, Ghiringhelli F, Tesniere A, Obeid M, Ortiz C, Criollo A (2007). Toll-like receptor 4-dependent contribution of the immune system to anticancer chemotherapy and radiotherapy. Nat Med.

[20] Michaud M, Martins I, Sukkurwala AQ, Adjemian S, Ma Y, Pellegatti P (2011). Autophagy-dependent anticancer immune responses induced by chemotherapeutic agents in mice. Science.

[21] Ma Y, Adjemian S, Mattarollo SR, Yamazaki T, Aymeric L, Yang H (2013). Anticancer chemotherapy-induced intratumoral recruitment and differentiation of antigen-presenting cells. Immunity.

[22] Parra ER, Uraoka N, Jiang M, Cook P, Gibbons D, Forget MA (2017). Validation of multiplex immunofluorescence panels using multispectral microscopy for immune-profiling of formalin-fixed and paraffin-embedded human tumor tissues. Sci Rep.

[23] Geisinger K, Moreira AL, Nicholson AG, Rami-Porta R, Travis WD. Lung cancer staging and grading. In: Travis WD, Brambilla E, Burke AP, Marx A, Nicholson AG, editors. WHO Classification of Tumours of the Lung, Pleura, Thymus and Heart. World Health Organization 2015; 4th Edition. p.14-15.

[24] Travis WD, Brambilla E, Noguchi M, Nicholson AG, Geisinger KR, Yatabe Y (2011). International association for the study of lung cancer/american thoracic society/european respiratory society international multidisciplinary classification of lung adenocarcinoma. J Thorac Oncol.

[25] Stack EC, Wang C, Roman KA, Hoyt CC (2014). Multiplexed immunohistochemistry, imaging, and quantitation: a review, with an assessment of Tyramide signal amplification, multispectral imaging and multiplex analysis. Methods.

[26] Stack EC, Foukas PG, Lee PP (2016). Multiplexed tissue biomarker imaging. J Immunother Cancer.

[27] Pataer A, Kalhor N, Correa AM, Raso MG, Erasmus JJ, Kim ES (2012). Histopathologic response criteria predict survival of patients with resected lung cancer after neoadjuvant chemotherapy. J Thorac Oncol.

[28] McDaniel AS, Alva A, Zhan T, Xiao H, Cao X, Gursky A (2016). Expression of PDL1 (B7-H1) before and after neoadjuvant chemotherapy in urothelial carcinoma. Eur Urol Focus.

[29] Katsuya Y, Horinouchi H, Asao T, Kitahara S, Goto Y, Kanda S (2016). Expression of programmed death 1 (PD-1) and its ligand (PD-L1) in thymic epithelial tumors: impact on treatment efficacy and alteration in expression after chemotherapy. Lung Cancer.

[30] Mesnage SJL, Auguste A, Genestie C, Dunant A, Pain E, Drusch F (2017). Neoadjuvant chemotherapy (NACT) increases immune infiltration and programmed death-ligand 1 (PD-L1) expression in epithelial ovarian cancer (EOC). Ann Oncol.

[31] Song Z, Yu X, Zhang Y (2016). Altered expression of programmed death-ligand 1 after neo-adjuvant chemotherapy in patients with lung squamous cell carcinoma. Lung Cancer.

[32] Leduc C, Adam J, Louvet E, Sourisseau T, Dorvault N, Bernard M (2018). TPF induction chemotherapy increases PD-L1 expression in tumour cells and immune cells in head and neck squamous cell carcinoma. ESMO Open.

[33] Hato SV, Khong A, de Vries IJ, Lesterhuis WJ (2014). Molecular pathways: the immunogenic effects of platinum-based chemotherapeutics. Clin Cancer Res.

[34] Kepp O, Senovilla L, Kroemer G (2014). Immunogenic cell death inducers as anticancer agents. Oncotarget.

[35] Gong W, Song Q, Lu X, Gong W, Zhao J, Min P (2011). Paclitaxel induced B7-H1 expression in cancer cells via the MAPK pathway. J Chemother.

[36] Zhang P, Su DM, Liang M, Fu J (2008). Chemopreventive agents induce programmed death-1-ligand 1 (PD-L1) surface expression in breast cancer cells and promote PD-L1-mediated T cell apoptosis. Mol Immunol.

[37] Peng J, Hamanishi J, Matsumura N, Abiko K, Murat K, Baba T (2015). Chemotherapy induces programmed cell death-ligand 1 overexpression via the nuclear factor-kappaB to Foster an immunosuppressive tumor microenvironment in ovarian Cancer. Cancer Res.

[38] Yang M, Liu P, Wang K, Glorieux C, Hu Y, Wen S (2017). Chemotherapy induces tumor immune evasion by upregulation of programmed cell death ligand 1 expression in bone marrow stromal cells. Mol Oncol.

[39] Ikeda S, Okamoto T, Okano S, Umemoto Y, Tagawa T, Morodomi Y (2016). PD-L1 is upregulated by simultaneous amplification of the PD-L1 and JAK2 genes in non-small cell lung Cancer. J Thorac Oncol.

[40] Banat GA, Tretyn A, Pullamsetti SS, Wilhelm J, Weigert A, Olesch C (2015). Immune and inflammatory cell composition of human lung Cancer stroma. PLoS One.

[41] Kammertoens T, Schuler T, Blankenstein T (2005). Immunotherapy: target the stroma to hit the tumor. Trends Mol Med.

[42] Spiotto MT, Schreiber H (2005). Rapid destruction of the tumor microenvironment by CTLs recognizing cancer-specific antigens cross-presented by stromal cells. Cancer Immun.

[43] Zhang B, Bowerman NA, Salama JK, Schmidt H, Spiotto MT, Schietinger A (2007). Induced sensitization of tumor stroma leads to eradication of established cancer by T cells. J Exp Med.

[44] Kroemer G, Galluzzi L, Zitvogel L (2013). Immunological effects of chemotherapy in spontaneous breast cancers. Oncoimmunology.

[45] Bourgeois C, Veiga-Fernandes H, Joret AM, Rocha B, Tanchot C (2002). CD8 lethargy in the absence of CD4 help. Eur J Immunol.

[46] Kurts C, Carbone FR, Barnden M, Blanas E, Allison J, Heath WR (1997). CD4+ T cell help impairs CD8+ T cell deletion induced by cross-presentation of self-antigens and favors autoimmunity. J Exp Med.

[47] Bevan MJ (2004). Helping the CD8(+) T-cell response. Nat Rev Immunol.

[48] Lopez-Verges S, Milush JM, Pandey S, York VA, Arakawa-Hoyt J, Pircher H (2010). CD57 defines a functionally distinct population of mature NK cells in the human CD56dimCD16+ NK-cell subset. Blood.

[49] Blankenstein T (2005). The role of tumor stroma in the interaction between tumor and immune system. Curr Opin Immunol.

[50] Sica A, Mantovani A (2012). Macrophage plasticity and polarization: in vivo veritas. J Clin Invest.

[51] Roth JA, Fossella F, Komaki R, Ryan MB, Putnam JB, Lee JS (1994). A randomized trial comparing perioperative chemotherapy and surgery with surgery alone in resectable stage IIIA non-small-cell lung cancer. J Natl Cancer Inst.

