# Supplementary material for: Effect of neoadjuvant chemotherapy on the immune microenvironment in non–small cell lung carcinomas as determined by multiplex immunofluorescence and image analysis approaches
Source: J Immunother Cancer. 2018 Jun 6;6:48. doi: 10.1186/s40425-018-0368-0 (PMC5989476; doi:10.1186/s40425-018-0368-0)
Supplement: Supplementary file 1 — Figure S1. Representative multiplex immunofluorescence (mIF) workflow, showing different steps in the process of analysis. (A) Vectra 3.0 multispectral microscope used for the analysis. (B) Phenochart 1.0.4 software showing an image scanning at ×10 and selection of five intratumoral areas (669×500 μm each) for further analysis. (C) mIF image scanning at ×20 and viewed in the InForm 2.1.3 image analysis software. (D) Composed image showing the different phenotypes with panel 1. (E) Tumor segmentation (epithelial and stromal compartments) using the InForm software. (E) Cell segmentation to characterize individual cells using DAPI as a counterstaining marker, and cell phenotyping identifying different subgroups of cells supervised by a pathologist. (PPTX 6362 kb) [file 40425_2018_368_MOESM1_ESM.pptx]

## Slide 1
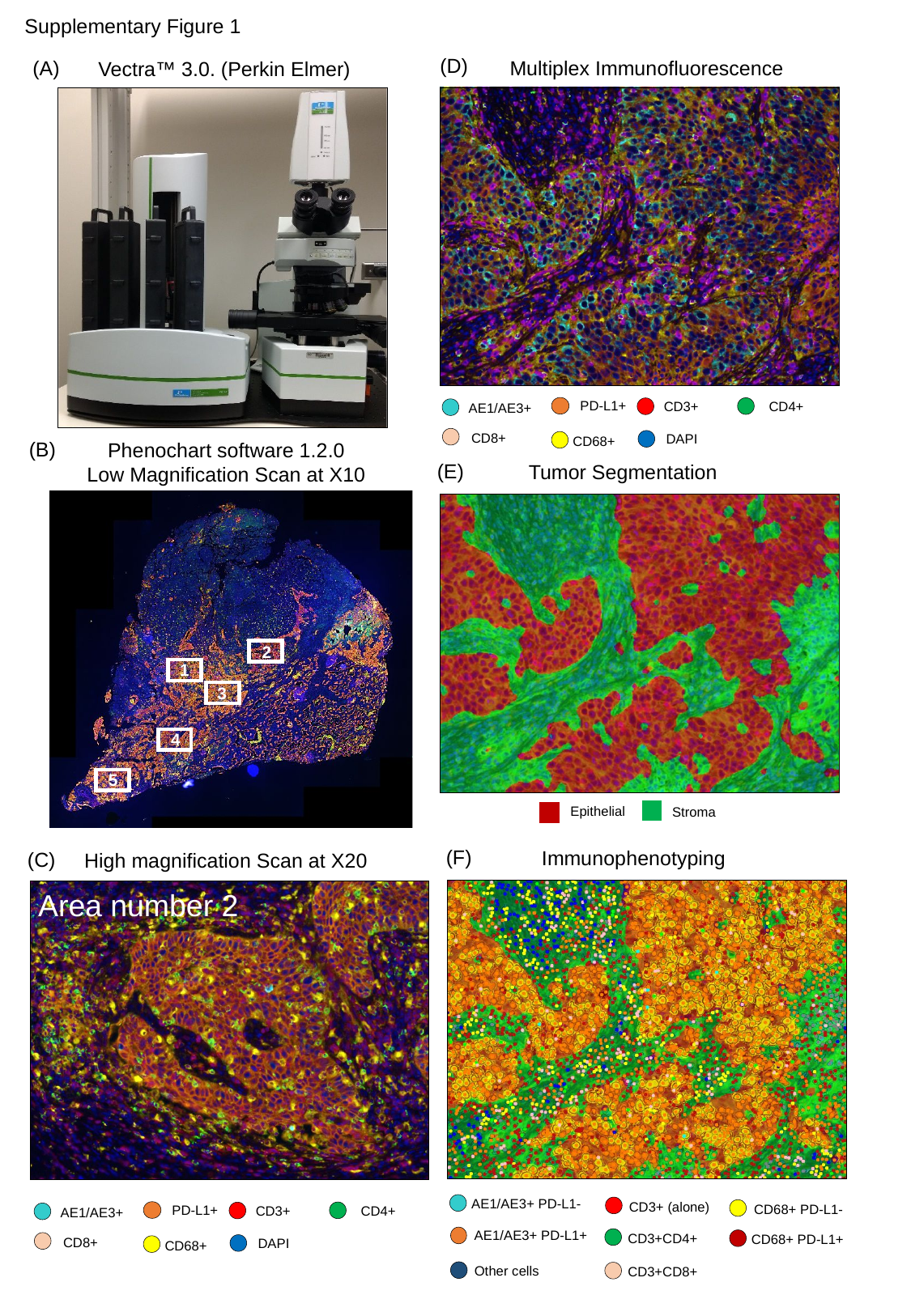

Supplementary Figure 1
(D)
(A)
Multiplex Immunofluorescence
Vectra™ 3.0. (Perkin Elmer)
PD-L1+
CD3+
CD4+
AE1/AE3+
CD8+
DAPI
CD68+
(B)
Phenochart software 1.2.0
Low Magnification Scan at X10
(E)
Tumor Segmentation
2
1
3
4
5
Epithelial
Stroma
(F)
Immunophenotyping
(C)
High magnification Scan at X20
Area number 2
AE1/AE3+ PD-L1-
CD3+ (alone)
CD68+ PD-L1-
PD-L1+
CD3+
CD4+
AE1/AE3+
AE1/AE3+ PD-L1+
CD3+CD4+
CD68+ PD-L1+
CD8+
DAPI
CD68+
Other cells
CD3+CD8+
