# Supplementary material for: Effect of neoadjuvant chemotherapy on the immune microenvironment in non–small cell lung carcinomas as determined by multiplex immunofluorescence and image analysis approaches
Source: J Immunother Cancer. 2018 Jun 6;6:48. doi: 10.1186/s40425-018-0368-0 (PMC5989476; doi:10.1186/s40425-018-0368-0)
Supplement: Supplementary file 4 — Figure S4. Multiplex immunofluorescence images showing densities of various tumor-associated immune cell phenotypes as determined by panel 1 and panel 2 markers from representative NSCLCs treated with neoaduvant chemotherapy (NCT) or not treated with NCT (non-NCT). Numbers of T lymphocytes (CD3+), helper T cells (CD3 + CD4+), tumor-associated macrophages (TAM; CD68+), activated natural killer cells (CD57 + granzyme B + CD45RO−), memory antigen experienced cells (CD45RO + PD-1+), and antigen experienced PD-1+ cells as well as PD-L1+ malignant cells were higher in the NCT group than in the non-NCT group. Images ×200. (PPTX 29362 kb) [file 40425_2018_368_MOESM4_ESM.pptx]

## Slide 1
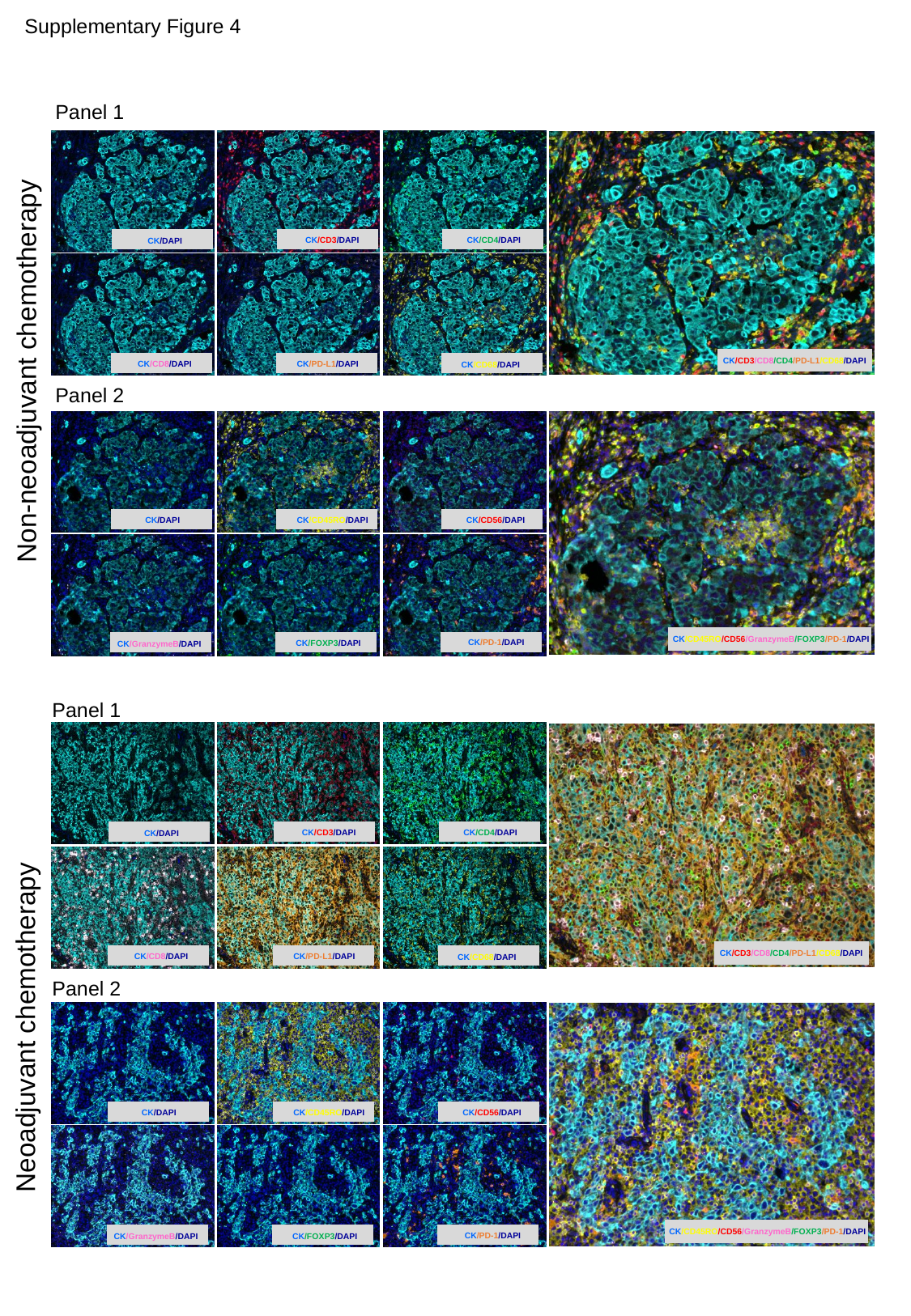

Supplementary Figure 4
Panel 1
CK/CD4/DAPI
CK/CD3/DAPI
CK/DAPI
Non-neoadjuvant chemotherapy
CK/CD3/CD8/CD4/PD-L1/CD68/DAPI
CK/CD8/DAPI
CK/PD-L1/DAPI
CK/CD68/DAPI
Panel 2
CK/CD45RO/DAPI
CK/CD56/DAPI
CK/DAPI
CK/CD45RO/CD56/GranzymeB/FOXP3/PD-1/DAPI
CK/PD-1/DAPI
CK/FOXP3/DAPI
CK/GranzymeB/DAPI
Panel 1
CK/CD4/DAPI
CK/CD3/DAPI
CK/DAPI
CK/CD3/CD8/CD4/PD-L1/CD68/DAPI
CK/CD8/DAPI
CK/PD-L1/DAPI
CK/CD68/DAPI
Panel 2
Neoadjuvant chemotherapy
CK/CD45RO/DAPI
CK/CD56/DAPI
CK/DAPI
CK/CD45RO/CD56/GranzymeB/FOXP3/PD-1/DAPI
CK/PD-1/DAPI
CK/FOXP3/DAPI
CK/GranzymeB/DAPI
