# Supplementary material for: Effect of neoadjuvant chemotherapy on the immune microenvironment in non–small cell lung carcinomas as determined by multiplex immunofluorescence and image analysis approaches
Source: J Immunother Cancer. 2018 Jun 6;6:48. doi: 10.1186/s40425-018-0368-0 (PMC5989476; doi:10.1186/s40425-018-0368-0)
Supplement: Supplementary file 5 — Table S1. Median densities of tumor-associated immune cells in NSCLCs of patients who received neoadjuvant chemotherapy (NCT) or did not receive NCT (non-NCT), by tumor compartment (N = 112) (DOCX 21 kb) [file 40425_2018_368_MOESM5_ESM.docx]

**Additional file 5 Table S1.** Median densities of tumor-associated immune cells in NSCLCs of patients who received neoadjuvant chemotherapy (NCT) or did not receive NCT (non-NCT), by tumor compartment (N=112)

| **Markers** | **NSCLC** | | ***P**** |
| --- | --- | --- | --- |
|  | **non-NCT (n=61)** | **NCT (n=51)** |  |
|  | Median Cell Density (cells/mm^2^) | |  |
| **Epithelial compartment** | |  |  |
| **Panel 1** | |  |  |
| CD3+ | 202.11 | 367.73 | **0.043** |
| CD3+CD4+ | 116.99 | 265.92 | **0.017** |
| CD3+CD8+ | 60.62 | 72.87 | 0.684 |
| CD68+ | 108.34 | 300.33 | **0.010** |
| CD68+PD-L1+ | 64.68 | 129.82 | **0.049** |
| **Panel 2** |  |  |  |
| CD45RO+ | 259.56 | 369.42 | 0.083 |
| CD45RO+CD57+granzymeB− | 103.79 | 256.50 | **0.006** |
| CD45RO+PD-1+ | 70.66 | 196.90 | **0.004** |
| CD45RO+FOXP3+ | 2.33 | 1.16 | 0.092 |
| CD57+granzyme B+CD45RO− | 5.56 | 20.29 | **0.006** |
| PD-1+ | 171.43 | 497.02 | **<0.001** |
| **Stromal compartment** |  |  |  |
| **Panel 1** |  |  |  |
| CD3+ | 1541.27 | 2233.70 | **0.029** |
| CD3+CD4+ | 1127.88 | 1946.92 | 0.067 |
| CD3+CD8+ | 257.84 | 441.03 | 0.476 |
| CD68+ | 446.86 | 712.07 | 0.180 |
| CD68+PD-L1+ | 278.13 | 418.88 | 0.292 |
| **Panel 2** |  |  |  |
| CD45RO+ | 4064.28 | 3661.30 | 0.290 |
| CD45RO+CD57+granzyme B− | 679.09 | 965.58 | 0.147 |
| CD45RO+PD-1+ | 569.54 | 693.73 | 0.191 |
| CD45RO+FOXP3+ | 35.80 | 25.53 | 0.060 |
| CD57+granzyme B+ CD45RO− | 10.41 | 28.27 | **0.002** |
| PD-1+ | 1451.99 | 2110.38 | 0.086 |

* Mann Whitney U test.
