# Supplementary material for: Effect of neoadjuvant chemotherapy on the immune microenvironment in non–small cell lung carcinomas as determined by multiplex immunofluorescence and image analysis approaches
Source: J Immunother Cancer. 2018 Jun 6;6:48. doi: 10.1186/s40425-018-0368-0 (PMC5989476; doi:10.1186/s40425-018-0368-0)
Supplement: Supplementary file 6 — Figure S5. Multiplex immunofluorescence images showing densities of various tumor-associated immune cell phenotypes (TAICs) as determined by panel 1 and panel 2 markers from the stromal and epithelial compartments of representative NSCLCs treated with neoadjuvant chemotherapy (NCT) or not treated with NCT (non-NCT). In general, densities of TAICs were higher in the stromal compartments than in their respective epithelial compartments in both NCT and non-NCT tumors. Overall, density of tumor-associated macrophages (TAMs; CD68+) was higher in NCT tumors than in non-NCT tumors, and density of memory/regulatory cells (CD45RO + FOXP3+) was lower in the NCT group than in the non-NCT group. Images ×200. (PPTX 15137 kb) [file 40425_2018_368_MOESM6_ESM.pptx]

## Slide 1
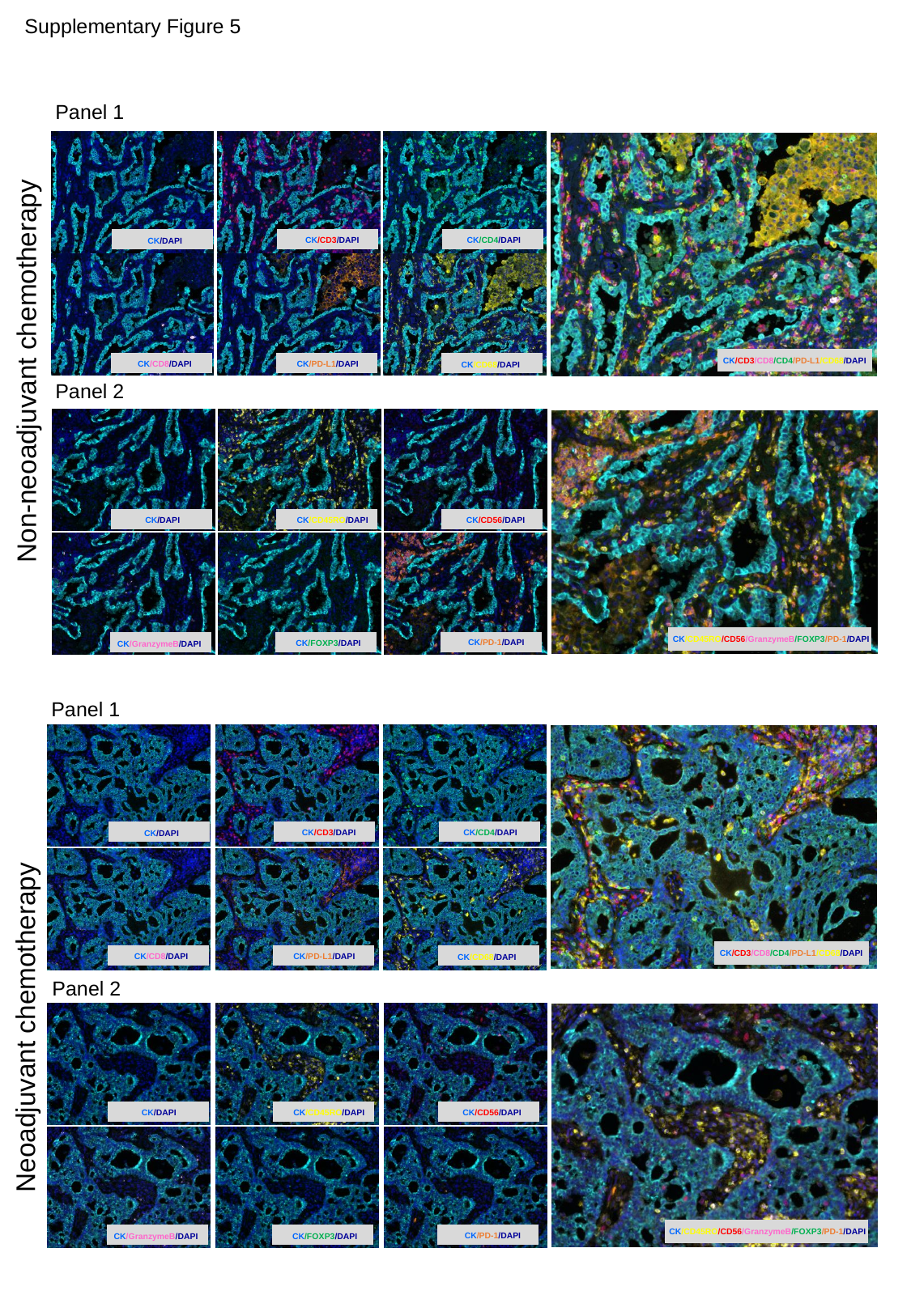

Supplementary Figure 5
Panel 1
CK/CD4/DAPI
CK/CD3/DAPI
CK/DAPI
Non-neoadjuvant chemotherapy
CK/CD3/CD8/CD4/PD-L1/CD68/DAPI
CK/CD8/DAPI
CK/PD-L1/DAPI
CK/CD68/DAPI
Panel 2
CK/CD45RO/DAPI
CK/CD56/DAPI
CK/DAPI
CK/CD45RO/CD56/GranzymeB/FOXP3/PD-1/DAPI
CK/PD-1/DAPI
CK/FOXP3/DAPI
CK/GranzymeB/DAPI
Panel 1
CK/CD4/DAPI
CK/CD3/DAPI
CK/DAPI
CK/CD3/CD8/CD4/PD-L1/CD68/DAPI
CK/CD8/DAPI
CK/PD-L1/DAPI
CK/CD68/DAPI
Panel 2
Neoadjuvant chemotherapy
CK/CD45RO/DAPI
CK/CD56/DAPI
CK/DAPI
CK/CD45RO/CD56/GranzymeB/FOXP3/PD-1/DAPI
CK/PD-1/DAPI
CK/FOXP3/DAPI
CK/GranzymeB/DAPI
