# Supplementary material for: Effect of neoadjuvant chemotherapy on the immune microenvironment in non–small cell lung carcinomas as determined by multiplex immunofluorescence and image analysis approaches
Source: J Immunother Cancer. 2018 Jun 6;6:48. doi: 10.1186/s40425-018-0368-0 (PMC5989476; doi:10.1186/s40425-018-0368-0)
Supplement: Supplementary file 7 — Table S2. Median densities of tumor-associated immune cells in NSCLCs from patients who received neoadjuvant chemotherapy (NCT) or did not receive NCT (non-NCT), by tumor compartment and histology (N = 112). (DOCX 23 kb) [file 40425_2018_368_MOESM7_ESM.docx]

**Additional file 7:Table S2**. Median densities of tumor-associated immune cells in NSCLCs from patients who received neoadjuvant chemotherapy (NCT) or did not receive NCT (non-NCT), by tumor compartment and histology (N=112)

| **Markers** | **ADC (n=61)** | | ***P**** | **SCC (n=51)** | | ***P**** |
| --- | --- | --- | --- | --- | --- | --- |
|  | **non-NCT** | **NCT** |  | **non-NCT** | **NCT** |  |
|  | Median Cell Density (cells/mm^2^) | |  | Median Cell Density (cells/mm^2^) | |  |
| **Epithelial compartment** |  |  |  |  |  |  |
| **Panel 1** |  |  |  |  |  |  |
| CD3+ | 317.21 | 343.91 | 0.640 | 155.41 | 799.41 | **0.023** |
| CD3+CD4+ | 172.16 | 237.79 | 0.176 | 92.22 | 699.47 | **0.019** |
| CD3+CD8+ | 94.64 | 64.82 | 0.380 | 44.10 | 100.25 | 0.695 |
| CD68+ | 212.39 | 358.71 | 0.208 | 74.07 | 262.85 | **0.016** |
| CD68+PD-L1+ | 82.80 | 149.00 | 0.377 | 31.23 | 76.49 | 0.134 |
| **Panel 2** |  |  |  |  |  |  |
| CD45RO+ | 263.30 | 359.91 | 0.337 | 248.92 | 404.44 | 0.090 |
| CD45RO+CD57+granzymeB− | 150.47 | 263.68 | 0.052 | 54.34 | 89.66 | 0.118 |
| CD45RO+PD-1+ | 103.70 | 205.90 | 0.052 | 46.75 | 84.04 | 0.070 |
| CD45RO+FOXP3+ | 3.48 | 1.30 | 0.085 | 0.00 | 0.84 | 0.329 |
| CD57+Granzyme B+CD45RO− | 2.95 | 24.76 | **0.001** | 7.81 | 6.85 | 0.833 |
| PD-1+ | 248.69 | 504.65 | 0.074 | 100.82 | 479.35 | **<0.001** |
| **Stromal compartment** |  |  |  |  |  |  |
| **Panel 1** |  |  |  |  |  |  |
| CD3+ | 1383.88 | 2151.06 | 0.126 | 1829.83 | 3240.05 | 0.055 |
| CD3+CD4+ | 836.64 | 1933.86 | 0.132 | 1700.66 | 2480.56 | 0.126 |
| CD3+CD8+ | 305.32 | 440.70 | 0.706 | 250.42 | 486.39 | 0.508 |
| CD68+ | 376.35 | 645.49 | 0.422 | 563.64 | 859.83 | 0.303 |
| CD68+PD-L1+ | 278.13 | 315.28 | 0.584 | 288.30 | 478.42 | 0.266 |
| **Panel 2** |  |  |  |  |  |  |
| CD45RO+ | 3088.66 | 1788.61 | 0.224 | 5088.42 | 4807.43 | 0.684 |
| CD45RO+CD57+granzymeB− | 649.45 | 868.21 | 0.276 | 772.02 | 1318.30 | 0.256 |
| CD45RO+PD-1+ | 609.30 | 661.91 | 0.457 | 535.91 | 991.48 | 0.221 |
| CD45RO+FOXP3+ | 54.17 | 16.55 | **0.001** | 27.64 | 36.47 | 0.417 |
| CD57+granzyme B+ CD45RO− | 7.01 | 43.71 | **0.001** | 15.16 | 18.55 | 0.525 |
| PD-1+ | 1810.64 | 1372.79 | 0.880 | 1232.11 | 2726.32 | **0.015** |

ADC, adenocarcinoma; SCC, squamous cell carcinoma

* Mann Whitney U test
