# Supplementary material for: Effect of neoadjuvant chemotherapy on the immune microenvironment in non–small cell lung carcinomas as determined by multiplex immunofluorescence and image analysis approaches
Source: J Immunother Cancer. 2018 Jun 6;6:48. doi: 10.1186/s40425-018-0368-0 (PMC5989476; doi:10.1186/s40425-018-0368-0)
Supplement: Supplementary file 8 — Table S3. Multivariate survival analysis (Cox regression model) of effects on survival of (A) tumor-associated macrophages (TAMs; CD68+) and (B) helper T cells (CD3 + CD4+) controlled by tumor stage in NSCLCs from patients who received neoadjuvant chemotherapy (N = 51). (DOCX 18 kb) [file 40425_2018_368_MOESM8_ESM.docx]

**Additional file 8: Table S3.** Multivariate survival analysis (Cox regression model) of effects on survival of (A) tumor-associated macrophages (TAMs; CD68+) and (B) helper T cells (CD3+CD4+) controlled by tumor stage in NSCLCs from patients who received neoadjuvant chemotherapy (N=51)

(A)

| **Overall Survival** |  |  |  |  |
| --- | --- | --- | --- | --- |
| **Variable** | **Categories** | **^±^HR** | **^◃^95% CI** | ****P*** |
| Epithelial/stromal TAMs CD68+ | Low vs High | 0.506 | 0.261-0.982 | 0.044 |
| Pathologic stage | III *vs* II | 1.828 | 0.545-6.133 | 0.329 |

(B)

| **Overall Survival** |  |  |  |  |
| --- | --- | --- | --- | --- |
| **Variable** | **Categories** | **^±^HR** | **^◃^95% CI** | ****P*** |
| Epithelial helper T cells CD3+CD4+ | Low vs High | 0.547 | 0.269-1.114 | 0.097 |
| Pathologic stage | III *vs* II | 1.443 | 0.404-5.156 | 0.572 |

HR= hazard ratio; CI: confidence interval
